# Comparison of setup accuracy of optical surface image versus orthogonal x‐ray images for VMAT of the left breast using deep‐inspiration breath‐hold

**DOI:** 10.1002/acm2.14117

**Published:** 2023-08-03

**Authors:** Wei Lu, Linda X. Hong, Nelson Yamada, Sean L. Berry, Yulin Song, Wookjin Choi, Laura I. Cerviño, Xiaoli Tang, James G. Mechalakos, Paul B. Romesser, Simon Powell, Guang Li

**Affiliations:** ^1^ Department of Medical Physics Memorial Sloan Kettering Cancer Center New York New York USA; ^2^ Department of Radiation Oncology Memorial Sloan Kettering Cancer Center New York New York USA

**Keywords:** 2DkV, CBCT, DIBH, left breast cancer, optical surface image, orthogonal x‐ray image, OSI, radiotherapy, setup accuracy

## Abstract

To compare the setup accuracy of optical surface image (OSI) versus orthogonal x‐ray images (2DkV) using cone beam computed tomography (CBCT) as ground truth for radiotherapy of left breast cancer in deep‐inspiration breath‐hold (DIBH). Ten left breast DIBH patients treated with volumetric modulated arc therapy (VMAT) were studied retrospectively. OSI, 2DkV, and CBCT were acquired weekly at treatment setup. OSI, 2DkV, and CBCT were registered to planning CT or planning DRR based on a breast surface region of interest (ROI), bony anatomy (chestwall and sternum), and both bony anatomy and breast surface, respectively. These registrations provided couch shifts for each imaging system. The setup errors, or the difference in couch shifts between OSI and CBCT were compared to those between 2DkV and CBCT. A second OSI was acquired during last beam delivery to evaluate intrafraction motion. The median absolute setup errors were (0.21, 0.27, 0.23 cm, 0.6°, 1.3°, 1.0°) for OSI, and (0.26, 0.24, 0.18 cm, 0.9°, 1.0°, 0.6°) for 2DkV in vertical, longitudinal and lateral translations, and in rotation, roll and pitch, respectively. None of the setup errors was significantly different between OSI and 2DkV. For both systems, the systematic and random setup errors were ≤0.6 cm and ≤1.5° in all directions. Nevertheless, larger setup errors were observed in some sessions in both systems. There was no correlation between OSI and CBCT whereas there was modest correlation between 2DkV and CBCT. The intrafraction motion in DIBH detected by OSI was small with median absolute translations <0.2 cm, and rotations ≤0.4°. Though OSI showed comparable and small setup errors as 2DkV, it showed no correlation with CBCT. We concluded that to achieve accurate setup for both bony anatomy and breast surface, daily 2DkV can't be omitted following OSI for left breast patients treated with DIBH VMAT.

## 1 INTRODUCTION

Radiation therapy (RT) is a common treatment modality for patients with breast cancer. There is some incidental radiation dose to the heart, especially for patients with left‐sided tumors. It was shown that the rates of major coronary events increased linearly with the mean dose to the heart by 7.4% per gray, with no apparent threshold.^1^ One effective approach to reduce the heart dose is to increase the distance between the chest wall and heart such as with deep‐inspiration breath‐hold (DIBH).^2^, ^3^, ^4^ A large number of studies showed that DIBH significantly reduced the dose to heart (by 25%−75%) as well as the dose to cardiac substructures,^3^, ^5^ which could potentially translate into the clinical benefit of reduced cardiac toxicity.^6^, ^7^ Secondary advantages of DIBH include reducing relative volume of lung exposed to radiation and minimizing respiratory motion.^2^, ^8^

Several systems have been developed to monitor patient breathing and guide DIBH treatment, including the widely used real‐time position management (RPM) system (Varian Medical Systems, Palo Alto, CA),^3^, ^9^, ^10^, ^11^, ^12^ and the recent 3D optical surface image (OSI) systems such as AlignRT (VisionRT, London, UK).^4^, ^13^, ^14^, ^15^, ^16^, ^17^, ^18^, ^19^, ^20^, ^21^, ^22^ In a previous comparison study, we found that the setup errors of chestwall were small and comparable with RPM‐ or OSI‐guided DIBH; while the setup errors of heart were smaller in tangential patients with OSI‐guided DIBH and comparable in VMAT patients.^23^ We attributed this advantage of OSI to its capability of monitoring the 3D motion of a much larger region versus monitoring only 1D motion of a small region with RPM. Another advantage of OSI is that it is not only a breathing monitoring system (like RPM) but also a 3D imaging system for setting up a patient to the planning position. The OSI‐guided setup allows align the arm first and then a breast region of interest (ROI) to minimize breast deformation and reproduce local lymph node positions.^24^ For RPM‐guided DIBH, an additional onboard x‐ray imaging system (MV portal imaging, 2DkV, or CBCT) is needed to setup a patient based on bony anatomy or lumpectomy cavity or breast tissue. For OSI‐guided DIBH, the OSI itself can be used to setup a patient, based on a 3D OSI of the patient and the breast ROI. Therefore, a practical question is whether one can omit onboard x‐ray imaging when OSI is used?

To answer the question, we compared the setup accuracy of 2DkV (our standard of care) versus OSI alone by using CBCT as the ground truth. There are several similar studies in the literature. Alderliesten et al. compared setup accuracy of OSI versus CBCT.^13^ Rossi et al. and Laaksomaa et al. compared setup errors using OSI only versus OSI + 2DkV.^17^, ^25^ Penninkhof et al. assessed the benefit of OSI versus tattoos using online CBCT registration.^26^ To the best of our knowledge, ours is the first study that examines all three imaging systems 2DkV, OSI, and CBCT in the same session, with patients presumably at the same position since no couch shifts or corrections were applied between imaging.

## 2 METHODS AND MATERIALS

### 2.1 Patients

This retrospective study was approved by our Institutional Review Board. Ten patients with left breast cancer treated with VMAT were evaluated. The cohort included 5 patients post mastectomy (2 with tissue expander), 4 patients post lumpectomy, and 1 patient with no surgery. All patients underwent whole breast (*n* = 5) or chest wall (*n* = 5) irradiation. The median age was 57 (range 32−74). All patients were coached and treated with RPM‐guided DIBH, while using OSI simultaneously for comparison purpose without interfere the DIBH guidance.

### 2.2 Simulation, setup, and breath‐hold monitoring

All patients were positioned with a CIVCO breast board (CIVCO Medical Solutions, Coralville, Iowa) with both arms above head and a roll under knees. Patients were scanned with 16‐slice Philips Brilliance CT scanners for treatment simulation. The RPM system was used to monitor patient breath and coach patient to perform DIBH at CT simulation and at treatment. The RPM reflective block was positioned close to the xiphoid and its position was tattooed on the patient at time of simulation. Prior to the simulation scan, patients were coached to follow a modified slow vital capacity maneuver—normal tidal breathing for a few cycles, then a deep inhale followed by a deep exhale, followed by a deep inhale which is held at a level close to peak inspiration (DIBH) for 15– 20 s. This was monitored with the RPM system and repeated a few times to familiarize patients with the process. For the planning CT scan, patients were coached into this maneuver and the DIBH treatment planning CT scan was acquired during the breath‐hold. Another free breathing (FB) CT scan was acquired immediately following the DIBH scan and patients were instructed to not move between the two scans. The FB scan was used for setup to skin tattoos and for backup in case the patient was unable to perform DIBH in treatment.

### 2.3 Contouring and treatment planning

The MIM (MIM Software Inc. Cleveland, OH) and the Eclipse (Varian Medical Systems, Palo Alto, CA) systems were used for contouring and treatment planning, respectively. Both contouring and planning were done on the DIBH CT scan. Structures were contoured by physicians (CTVs, PTVs, and heart), and by planners (contralateral breast, lungs, and esophagus). Breast and regional nodes (internal mammary, supraclavicular, and axillary lymph nodes) were treated with four or five partial arcs (60–100 degrees), which were planned and optimized with Eclipse VMAT optimization.^27^ 6MV and a bolus (0.3 cm thick) covering the entire breast/chestwall area was used to assure adequate skin dose for all patients. Elasto‐Gel bolus (Pro Therapy Supplies, Duluth, GA) with built‐in white fabric on the exterior side was used to reflect the light for OSI. Finally, the upper and lower gating threshold lines were set to 0.25 cm above and below the average level of the breath‐hold at simulation. This defined a gating window of 0.5 cm.

### 2.4 Setup imaging and data analysis

**FIGURE 1:**
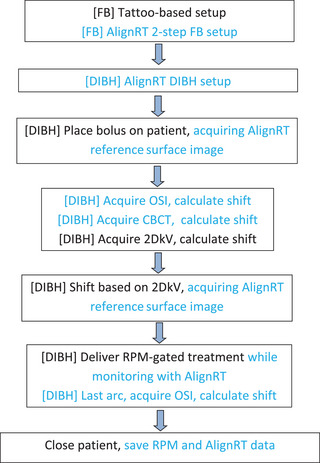
Flow chart. Black text indicates standard of care and blue text indicates additional procedure. All deep‐inspiration breath‐hold (DIBHs) were coached using the real‐time position management (RPM) system, while monitored using the AlignRT system.

Patients were treated on a Varian True Beam (Varian Medical Systems, Palo Alto, CA). The OSI system utilized AlignRT (Version 5.1) with three cameras. A breast surface ROI that covered the entire ipsilateral breast/chest wall and half of the contralateral breast was created in AlignRT for FB and DIBH, respectively.^24^ Figure 1 illustrates the workflow for this evaluation study, a more detailed description of the AlignRT workflow is presented in.^24^ Patients were first setup based on skin tattoos and shifted to isocenter in FB. Then a two‐step FB setup, guided by the in‐room AlignRT, was applied: (1) to align the arm and chin first by adjusting the patient, and (2) to align the FB breast ROI—while AlignRT monitoring was on, couch was shifted so that all real time deltas (RTDs) became green, that is, within tolerance, which were set to ≤0.3 cm in vertical, longitudinal and lateral (VRT, LNG, LAT) translations, and ≤3.0° in rotation, roll and pitch (RTN, ROL, PIT), respectively. After the FB setup, the DIBH breast ROI was selected, and patient was coached to perform a DIBH while the couch was shifted (except that VRT was kept unchanged) so that all RTDs were green again. This could be repeated for another DIBH to check reproducibility. We then placed sticky bolus on patient, coached patient to perform a DIBH while acquiring a new AlignRT reference surface image. Next, we coached patient to perform DIBH and acquired simultaneously an OSI and an CBCT during DIBH. The OSI was matched to the latest reference based on the DIBH breast ROI. The resulting 6D couch shifts were recorded but not applied. The CBCT used a “Full Fan” protocol with a gantry rotation of 200° over ∼40 s. Some patients needed more than one DIBH cycle to complete the CBCT acquisition. The CBCT was matched to the planning CT on the True Beam system using Auto Match followed by manual adjustment based on both bony anatomy (anterior sternum and chestwall ribcage) and the breast surface. Again the resulting 6D couch shifts were recorded but not applied. Next, we coached patient to perform two DIBHs and acquired 2DkV in anterior‐posterior (AP) and left lateral (LAT) directions respectively. The 2DkV were matched to the planning digitally reconstructed radiographs (DRRs) based on bony anatomy. The resulting 5D couch shifts (roll was not calculated and all roll values were set to 0°) were recorded and applied while patient was coached to perform a DIBH. Immediately after the couch were shifted and within the same DIBH, another AlignRT reference surface image was acquired. Finally, the treatment was delivered using RPM‐guided DIBH by following our standard of care, while AlignRT was used simultaneously to monitor patient's surface motion. During the last arc delivery, an end‐of‐delivery OSI was acquired for evaluating intrafraction motion in DIBH. The process of applying couch shifts based on 2DkV is our standard of care and was performed daily whereas the process of AlignRT/OSI and CBCT was for observation only and performed only once a week.

The setup errors were calculated as the differences in couch shifts between OSI and CBCT, and between 2DkV and CBCT, respectively, using CBCT as the ground truth. We calculated the group mean (*M*), systematic error (*Σ*), and random error (*σ*) to assess the difference in setup errors between OSI and 2DkV. The rank sum test was used to compare the two systems statistically with a significance level of 0.05. Finally, we ran linear regression analysis to assess the correlations in couch shifts between OSI and CBCT, and between 2DkV and CBCT, respectively. The coefficient of determination (*R* ^2^, where R denotes the correlation coefficient) was reported.

## 3 RESULTS

**FIGURE 2:**
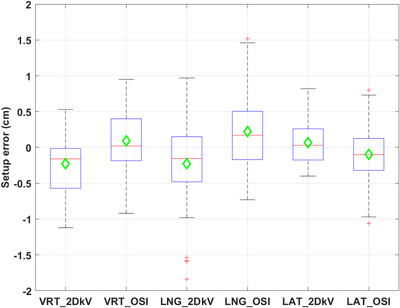
Box plots of the translational setup errors (difference from cone beam computed tomography [CBCT]) for 2DkV and optical surface image (OSI). These symbols are the same in all boxplots: the central line/marker indicates the median/mean respectively, the box indicates the 25th and 75th percentiles, the length of whisker is 1.5 times the interquartile range, the “+” is considered as outlier.

**FIGURE 3:**
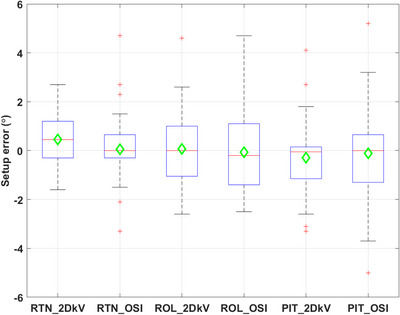
Box plots of the rotational setup errors (difference from cone beam computed tomography [CBCT]) for 2DkV and optical surface image (OSI).

Each patient had three to five weekly imaging sessions during which all three types of images were acquired. This resulted in a total of 40 imaging sessions for evaluation. Figures 2, 3 show the box plots of the setup errors for 2DkV and OSI. The median absolute translational setup errors in (VRT, LNG, LAT) were (0.21, 0.27, 0.23 cm) for OSI and (0.26, 0.24, 0.18 cm) for 2DkV, with *p*‐values (0.86, 0.87, 0.40). The median absolute rotational setup errors in (RTN, ROL, PIT) were (0.6°, 1.3°, 1.0°) for OSI and (0.9°, 1.0°, 0.6°) for 2DkV, with *p*‐values (0.21, 0.50, 0.33). Tables 1 and 2 show the *M*, *Σ*, and *σ* for these setup errors.

**TABLE 1:** Setup errors of OSI and its correlation with CBCT.

	Translation (cm)			Rotation (°)		
	VRT	LNG	LAT	RTN	ROL	PIT
*M*	0.11	0.25	−0.09	0.1	0.0	−0.1
*Σ*	0.29	0.32	0.32	1.0	1.2	1.5
*σ*	0.33	0.47	0.35	1.2	1.5	1.5
*R* ^2^	0.02	0.02	0.01	0.10	0.002	0.08

Abbreviations: LAT, lateral; LNG, longitudinal; PIT, pitch; ROL, roll; RTN, rotation; VRT, vertical.

**TABLE 2:** Setup errors of 2DkV and its correlation with CBCT.

	Translation (cm)			Rotation (°)		
	VRT	LNG	LAT	RTN	ROL^a^	PIT
*M*	−0.21	−0.24	0.08	0.4	0.2	−0.3
*Σ*	0.35	0.57	0.23	0.4	1.3	0.9
*σ*	0.26	0.33	0.24	1.0	1.3	1.4
*R* ^2^	0.24	0.34	0.75	0.57	N/A	0.25

Abbreviations: LAT, lateral; LNG, longitudinal; PIT, pitch; ROL, roll; RTN, rotation; VRT, vertical.

^a^ ROL was not calculated and all ROL values were set to 0°.

**FIGURE 4:**
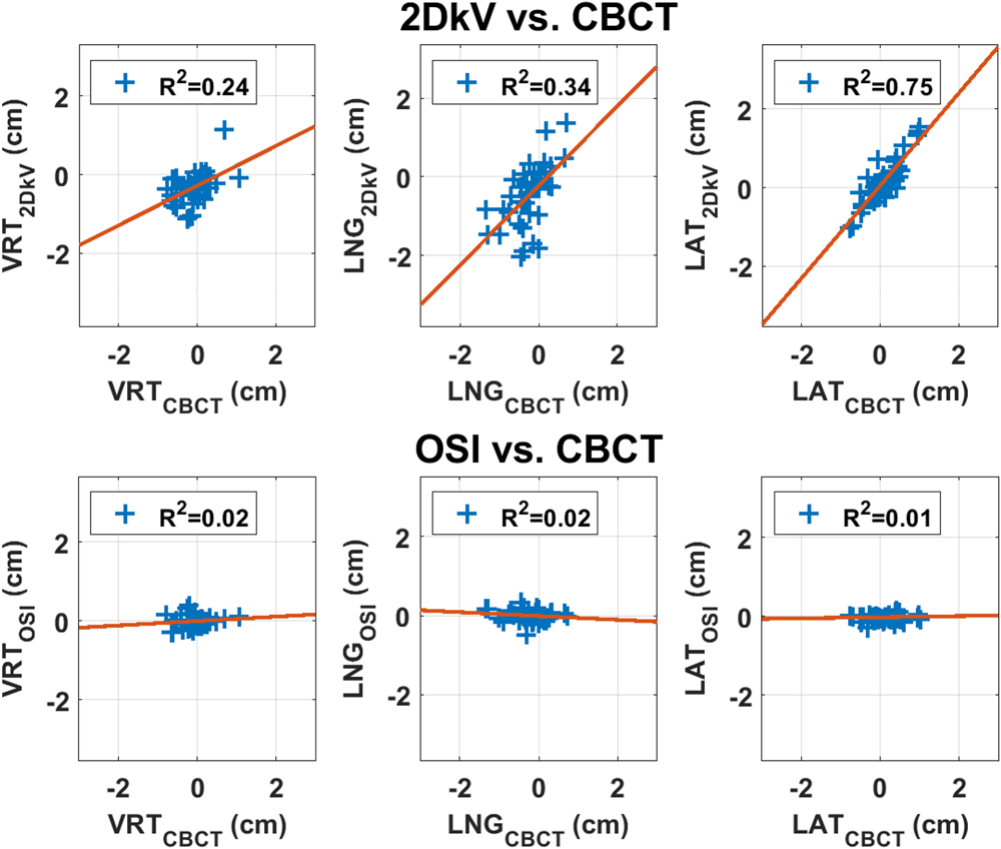
The scatter plots with regression lines of the translational couch shifts calculated by 2DkV and cone beam computed tomography (CBCT), and optical surface image (OSI), and CBCT.

**FIGURE 5:**
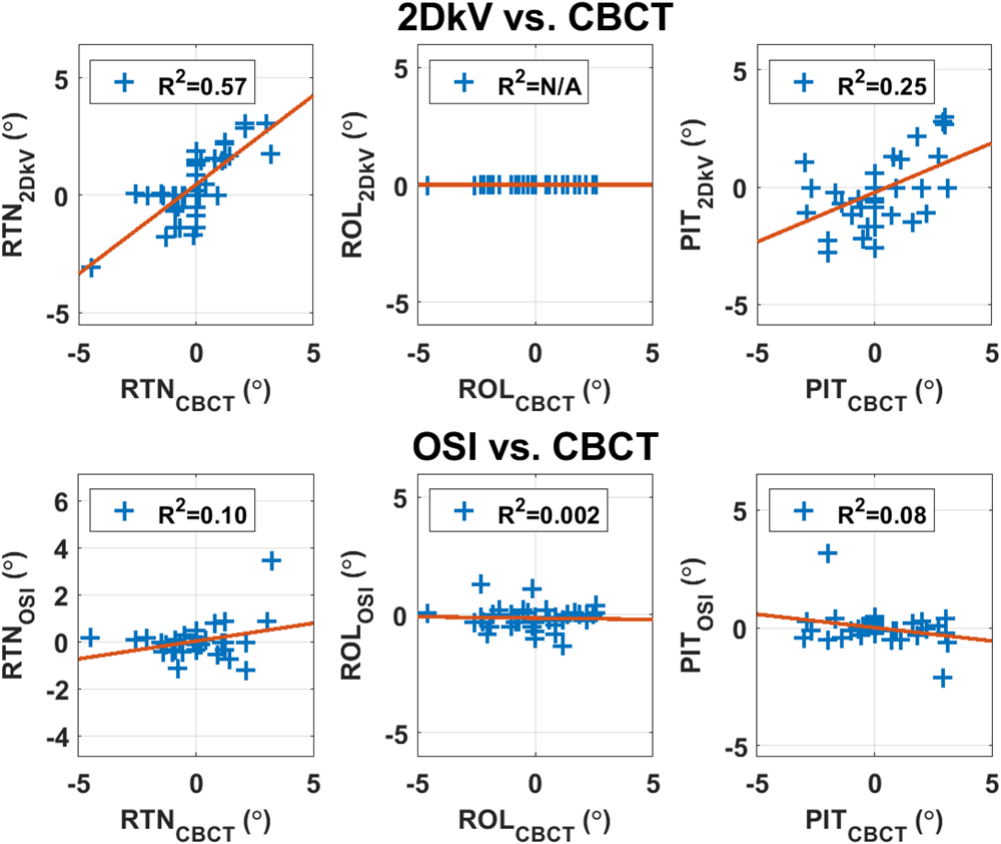
The scatter plots with regression lines of the rotational couch shifts calculated by 2DkV and cone beam computed tomography (CBCT), and optical surface image (OSI), and CBCT. For 2DkV, ROL was not calculated and all ROL values were set to 0°.

Figures 4, 5 compare the scatter plots with regression lines of the couch shifts calculated by 2DkV and CBCT with those calculated by OSI and CBCT. The coefficient of determination *R* ^2^ was shown in the legends and reported in Tables 1 and 2.

**FIGURE 6:**
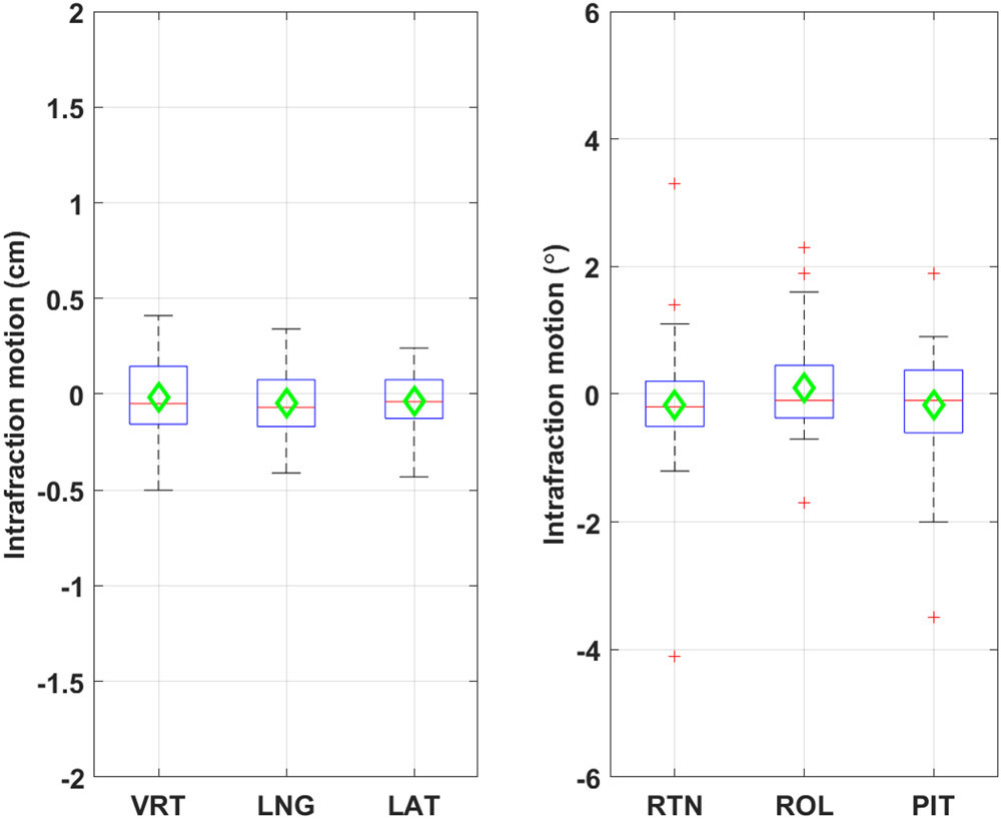
Box plots for the intrafraction motion in deep‐inspiration breath‐hold (DIBH) monitored by optical surface image (OSI).

Figure 6 shows the box plot for the intrafraction motion in DIBH, which are the differences between the couch shifts calculated by the end‐of‐delivery OSI and the OSI acquired immediately before the first beam delivery. The median absolute intrafraction translations were (0.16, 0.16, 0.10 cm) in (VRT, LNG, LAT), and rotations (0.4°, 0.4°, 0.4°) in (RTN, ROL, PIT), respectively. There were no statistically significant differences between the two OSIs (*p*‐values were 0.81, 0.17, 0.08, 0.07, 0.56, and 0.31, respectively). These results suggested that generally the intrafraction motions measured by the two OSIs were clinically insignificant and the breast ROI was stable during DIBHs within the same treatment session. Nevertheless, there were a few outliers in rotations and we can't infer any information for the intrafraction motions of internal anatomy.

## 4 DISCUSSIONS

The systematic and random translational setup errors (*Σ* and *σ*) ranged from 0.29 to 0.47 cm for OSI, and from 0.23 to 0.57 cm for 2DkV. These values fell in the reported range of *Σ* = 0.07 to 0.57 cm and *σ* = 0.09 to 0.73 cm, respectively.^28^ The systematic and random rotational setup errors ranged from 1.2 to 1.5° for OSI, and from 0.9 to 1.4° for 2DkV. There was no statistically significant difference between OSI and 2DkV in any translational or rotational setup errors (all *p*‐values > 0.05). In a review article of setup error in breast RT using CBCT, Batumalai et al. found that there was no clear relationship between the systematic and random setup errors detected and method of image registration between CBCT and CT, which include bony registration, surface registration, bony + surface registration, and surgical clips registration.^28^ That finding aligned with our results that the systematic and random setup errors were comparable between OSI (surface registration) and 2DkV (bony registration).

**FIGURE 7:**
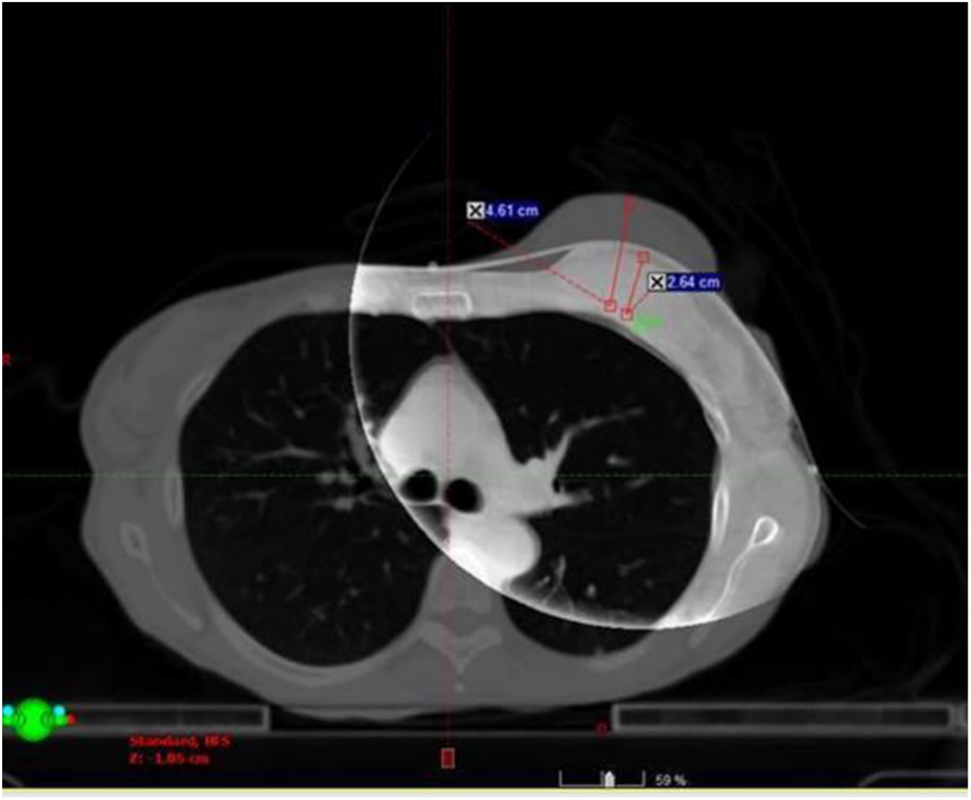
Cone beam computed tomography (CBCT) detected a clinically relevant change in breast tissue (2.0 cm) in an atypical patient with no surgery.

For both OSI and 2DkV, the median absolute setup errors were small, however, large setup errors were observed in some sessions—translational setup error > 1.0 cm for OSI in 3 sessions and for 2DkV in 5 sessions respectively, rotational setup error > 4.0° for OSI in 4 sessions and for 2DkV in 1 session respectively. One translation > 1.0 cm (LNG) and two rotations > 4.0° in OSI (RTN and ROL) and the only rotation > 4.0° in 2DkV (ROL) were all from a single patient, who had no surgery and had a drastic shrinkage (2.0 cm) of breast tissue (see Figure 7 and details below). It was likely that the large breast shrinkage led to these large setup errors. Four translations > 1.0 cm in 2DkV (all in LNG between −1.8 and −1.5 cm) were from another single patient, who had the most difficulty in following breathing instructions (patient has neurological disorder) and showed the greatest breathing variations (baseline drift, overshooting when taking a breath‐hold, seen in RPM trace). Other large translational or rotational setup errors were from different patients.

2DkV used 5D registration where roll was not calculated, and all roll values were set to 0°. Nevertheless, the systematic and random setup errors in roll were comparable to those of the OSI using 6D registration. Also, there was no correlation in roll between OSI and CBCT (*R* ^2^ 0.002). The difference in degrees of freedom did not seem to affect our conclusions.

Alderliesten et al. compared setup accuracy of OSI versus CBCT in 20 patients treated with DIBH RT post lumpectomy, using CBCT as gold standard.^13^ Though bony registration was used for matching CBCT to CT for online correction, surface registration was used offline for both matching OSI to CT and matching CBCT to CT for their reported results. The systematic and random errors between OSI and CBCT were smaller in translations (*Σ* 0.15–0.17 cm and *σ* 0.12–0.15 cm) but larger in rotations (likely due to the use of a single camera in their OSI, *Σ* 1.2–2.0° and *σ* 0.9–2.0°) compared to our results (Table 1), where surface registration was used for matching OSI to CT and bony + breast registration for matching CBCT to CT, respectively. Batin et al. compared setup accuracy of OSI with surface registration (10 patients) versus 2DkV with bony registration (5 patients) in postmastectomy patients treated with FB RT.^29^ A second OSI acquired immediately before beam on was compared to the planning CT to compute residual setup errors. OSI showed smaller residual setup errors (*Σ* 0.08–0.15 cm and *σ* 0.12–0.14 cm) than 2DkV (*Σ* 0.26–0.32 cm and *σ* 0.20–0.30 cm). However, computing the residual setup errors using the second OSI might have a favorable bias towards OSI. Cravo et al. compared setup accuracy of OSI versus skin tattoos in 20 patients (10 patients in each group) treated with whole breast FB RT, using CBCT as gold standard.^30^ They showed that OSI had smaller setup error (*Σ* 0.13–0.29 cm and *σ* 0.18–0.27 cm) than tattoos (*Σ* 0.29–0.33 cm and *σ* 0.24–0.31 cm). Topolnjak compared setup accuracy of MV portal image versus CBCT in 20 patients treated with tangential fields.^31^ They found that CBCT showed smaller residual setup errors (*Σ* 0.14–0.17 cm and *σ* 0.26–0.31 cm) than MV portal image (*Σ* 0.22–0.33 cm and *σ* 0.29–0.29 cm). They concluded that MV portal images underestimated the bony anatomy setup errors compared to CBCT, and that CBCT can be used as the gold standard.

Alderliesten et al. reported good correlations for translations (*R* ^2^ 0.70–0.90) and poor correlations for rotations (*R* ^2^ 0.19–0.37) between OSI and CBCT.^13^ We did not observe any correlations between OSI and CBCT (*R* ^2^ ≤ 0.02 for translations and *R* ^2^ ≤ 0.10 for rotations, Table 1 and Figures 4, 5). The main reason was that different registration methods were used in our study: surface registration for OSI to CT, and bony + breast registration for CBCT to CT, whereas Alderliesten et al. used surface registration offline for both OSI to CT and CBCT to CT. In our study, 2DkV showed higher correlations with CBCT (*R* ^2^ = 0.24–0.75 for translations and *R* ^2^ = 0.25–0.57 for rotations, Table 2 and Figures 4, 5), since bony registration was used for both. Another possible reason could be that the presence of bolus could potentially reduce the correlation between the OSI‐measured surface motion and CBCT‐measured bony anatomy motion due to patient movement during bolus placement, lack of conformation of bolus to the body surface, and/or bolus moving and deforming differently than body surface as the patient progresses through the breathing cycle.

With 3D technique, breast setup is checked with weekly MV imaging only and on daily basis with light field check without verification of isocenter position. With VMAT, however, the location of isocenter is important due to arc treatment and isocenter position has always been verified/corrected with 2D kV daily at our institution.^27^ Our results indicate that there was no correlation between OSI and CBCT whereas there was modest correlation between 2DkV and CBCT. 2DkV setup was based on bony anatomy whereas OSI setup was based on the surface. CBCT setup balanced the positional errors of bony anatomy and the breast surface. Since it's important in VMAT to achieve accurate setup for both bony anatomy and the breast surface, we conclude that daily 2DkV setup can't be omitted despite an initial OSI setup, based on results from this small cohort. Further work is in progress to analyze the residual errors of OSI in 2DkV in a larger cohort. Similarly, Alderliesten et al. did not recommend solely the use of OSI due to the challenge to distinguish whether a setup error in the AP direction is caused by anatomic changes or by a change in breath‐hold.^13^ Rossi et al. analyzed the positional errors of OSI with and without 2DkV image‐guidance during DIBH RT for 51 patients with either mastectomy or with N + conserving surgery.^25^ They measured the position errors of bony landmarks, the breath‐hold level, the breast surface, and the heart in 2DkV following OSI setup, and then in tangential kV image following 2DkV setup. Statistically significant improvement was observed with 2DkV setup in systematic and/or random errors for the ribs, th1 (surrogate marker for the axillary lymph nodes), spine, and the breast surface. More importantly, 2DkV setup significantly reduced the percentage of large positional errors exceeding 3‐ or 5‐mm with OSI setup only. They drew the same conclusion as ours that the 2DkV setup cannot be omitted despite OSI setup. Penninkhof et al. assessed the benefit of OSI (47 patients) versus tattoos (25 patients) using online CBCT registration.^26^ They found that the OSI setup reduced the total imaging time but did not reduce the observed setup errors in CBCT. They concluded similarly that daily online imaging is required following OSI setup to verify consistency of the internal anatomy for accurate breast RT using DIBH. Laaksomaa et al. found that 2DkV reduced the systematic setup errors from 0.3 cm with OSI only to 0.2 cm with OSI + 2DkV for whole‐breast DIBH treated with tangential fields.^17^ However, they questioned the clinical importance of this improvement and concluded that the frequency of 2DkV may be considerably reduced. They did not examine the correlations between OSI and 2DkV.

For this study, the 2DkV acquisition was automatically triggered when a patient reached the lower RPM gating threshold line, which was 0.25 cm below the average or middle breath‐hold level. After the study, our clinic added a 0.5 s delay before triggering the 2DkV, so that the images may be acquired closer to the middle breath‐hold level.^23^ We expect that would further increase the correlation between 2DkV and CBCT, as well as improve the setup accuracy of 2DkV. Our clinic has transitioned from RPM‐guided to OSI‐guided DIBH with daily 2DkV for these patients.^24^ The 2DkV acquisition is manually triggered by therapists after they coach a patient to perform a DIBH and observe that all RTDs on the OSI system are green. This practice has some inherent delay and thus the 2DkV is triggered closer to the middle breath‐hold level.

CBCT detected a clinically relevant shrinkage (about 2.0 cm) in breast tissue (Figure 7) in an atypical patient, who was treated with no surgery—unlike most patients were treated post‐surgery. The patient had triple negative, poorly differentiated invasive ductal carcinoma of the left breast with metastasis to the right lung. This shrinkage was not detected by OSI since OSI was matched to the reference CT based only on the surface with no internal anatomy information. It was not detected by 2DkV either since only bony anatomy was visible in 2DkV. It resulted in large setup errors for both OSI and 2DkV. To compensate for this shrinkage, the patient was re‐simulated and re‐planned. We recommend using CBCT at least twice a week to detect any clinically relevant changes in anatomy, such as for patients where there is concern for changes in normal tissue anatomy and/or rapid tumor response. The latest version of OSI (AlignRT V6.3) includes a surface deformation module that can be used to quantify the extent and amplitude of surface changes.^32^ Sorgato et al. showed its usefulness in detecting oedema in a breast phantom study,^32^ its potential to detect clinically relevant changes in patients is yet to be seen.

## 5 CONCLUSION

Compared to CBCT, OSI, and 2DkV had comparable and small setup errors in most left‐breast patients treated with DIBH VMAT. However, OSI did not show correlation with CBCT whereas 2DkV showed moderate correlation with CBCT. To achieve accurate setup for both bony anatomy and breast surface, daily 2DkV setup can't be omitted despite an initial OSI setup for left breast patients treated with DIBH VMAT.

## AUTHOR CONTRIBUTIONS

Lu had full access to all of the data in the study and takes responsibility of the integrity of the data and the accuracy of the data analysis. Concept and design: Lu, Li, Hong, Romesser, Powell, Tang, and Mechalakos. Acquisition, analysis, or interpretation of data: Lu, Yamada, Choi, Hong, Romesser, Li, Berry, Cerviño, and Tang. Drafting of the manuscript: Lu, Yamada, and Berry. Critical revision of the manuscript for important intellectual content: Lu, Hong, Li, Romesser, Berry, Cerviño, Song, and Mechalakos. Statistical analysis: Lu, Hong, Li, and Song. Administrative, technical, or material support: Li, Berry, and Powell.

## CONFLICT OF INTEREST STATEMENT

P.B.R received research funding (2019) and serves as a consultant for EMD Serono (2018‐present), receives research funding from XRAD Therapeutics (2022‐present), is a consultant for Faeth Therapeutics (2022‐present), is a consultant for Natera (2022‐present), and is a volunteer on the advisory board for the HPV Alliance and Anal Cancer Foundation non‐profit organizations.
